# Global Variation in Predictors of Uptake of Conservative Kidney Management: A Systematic Review and Meta‐Analysis

**DOI:** 10.1111/jorc.70071

**Published:** 2026-07-04

**Authors:** Kerry‐Lee Rosenberg, Naaheed Mukadam, Gordon Paterson, Jemima Sneddon, Diane Xu, Kashif Anwari, Aine Burns, Ben Caplin

**Affiliations:** ^1^ University College London London UK; ^2^ Royal Free Hospital NHS Foundation Trust London UK

**Keywords:** conservative kidney management, end‐stage kidney disease, kidney replacement therapy, meta‐analysis, social determinants of health

## Abstract

**Background:**

For older people and those with more comorbidities, kidney replacement therapy may not offer a mortality benefit. Conservative kidney management represents an alternative treatment pathway for kidney failure without dialysis.

**Objectives:**

To identify the clinical and socioeconomic factors associated with choosing a non‐dialysis care pathway and to explore geographical variation.

**Methods:**

MEDLINE, Embase and Web of Science databases were searched. Studies, which included patients with kidney failure choosing non‐dialysis care and a comparator group of patients preparing for or receiving kidney replacement therapy, were selected. Exposures included clinical and socioeconomic factors. The outcome was a choice to have non‐dialysis care. Random‐effects meta‐analysis was performed; with subgroup analysis by region.

**Results:**

In total 43 studies, including 51,872 participants, were selected. Female sex was associated with choosing non‐dialysis care (pooled OR 1.47, 95% CI 1.26–1.71). There was no overall association between non‐white ethnicity and treatment choice in Western countries. However, in North America non‐White groups had lower odds of receiving non‐dialysis care compared to white patients (OR 0.70, 95% CI 0.60–0.81). Socioeconomic deprivation was associated with choosing non‐dialysis care in Asia, as was low educational attainment (OR 2.85, 95% CI 1.54–5.26). There was an overall association between living alone and non‐dialysis care (pooled OR 1.55, 95% CI 1.24–1.93).

**Conclusion:**

The geographical variation in these results highlights the importance of social and political context in understanding treatment access. We identify living alone and low socioeconomic status as possible predictors of choosing non‐dialysis care. This analysis cannot ascertain if these associations arise from appropriate shared decisions, challenges to the decision making process or barriers to accessing kidney replacement therapy. Nevertheless, this review illustrates the interplay of socioeconomic, interpersonal and systemic factors at play and lays the foundation to unravelling these complexities.

## 1 Introduction

The prevalence of chronic kidney disease (CKD) is increasing globally (Chesnaye et al. 2024; GBD Chronic Kidney Disease Collaboration 2020), and the population reaching kidney failure (KF) is aging and becoming increasingly multimorbid and medically complex (Chesnaye et al. 2024; Tonelli et al. 2018). Kidney replacement therapy (KRT), in the form of dialysis or kidney transplant, remains the mainstay of treatment for KF. For older, more frail patients and those with more comorbidities, however, this option may not offer a survival or quality of life advantage (Brown et al. 2015; Carson et al. 2009; Chandna et al. 2011; Hussain et al. 2013; Murtagh et al. 2007). In addition, KRT is a burdensome treatment and is associated with increased rates of hospitalisation (Carson et al. 2009; Verberne et al. 2020), higher intensity of treatment at end of life (Tam‐Tham et al. 2020), and reduced uptake of palliative care (Hussain et al. 2013) and hospice(Carson et al. 2009). Conservative kidney management (CKM) provides an alternative treatment pathway, which may better serve the priorities of some patients. CKM is defined as ‘planned, holistic, patient‐centred care' for patients with stage 5 CKD, without dialysis. It includes management of the symptoms and complications of advanced CKD and interventions to slow progression, as well as multidisciplinary psychological and social support (Davison et al. 2015).

Treatment decisions in KF should ideally be made through a shared decision‐making process, which takes into account both clinician recommendation and the personal priorities of the patient (Kanbay et al. 2024). These decisions are often complex, however, and may be influenced by socioeconomic, cultural and wider systemic factors (Shi et al. 2022). Furthermore, availability of KRT and provision of CKM programmes vary widely around the world (Hole et al. 2024). Predictors of uptake of CKM may, therefore, be influenced by both barriers to KRT access and sociocultural aspects of the shared‐decision making process. The challenge is in understanding whether an association arises from appropriate and holistic shared decision‐making or whether it represents barriers either to accessing KRT or engaging with a potentially beneficial decision to have CKM. The latter may represent modifiable factors. Identifying and describing these associations is an essential first step in untangling these complexities and may inform more focused research in future.

This systematic review and meta‐analysis aims firstly to identify the demographic, clinical and socioeconomic factors associated with choosing CKM rather than KRT. Secondly, it aims to explore geographical variation in the predictors of uptake of CKM. As cautioned above, the provision of CKM is highly variable across geographical areas, and not all countries are able to offer a formalised CKM programme. The term non‐dialysis care is, therefore, used as a substitute throughout this report (the exception being when specific studies with defined CKM cohorts are referenced). Finally, whilst we refer to treatment choices throughout, we acknowledge that multiple factors influence what treatment is ultimately received (including accessibility, medical opinion and advice, family involvement and clinical uncertainty). The term ‘choice’ may not capture the underlying complexity of these decisions.

## 2 Methods

This systematic review and meta‐analysis is registered on the PROSPERO database (CRD42025628123) and is reported according to the Preferred Reporting Items for Systematic reviews and Meta‐Analyses (PRISMA) guidelines (Page et al. 2021) (checklist in File S1).

### 2.1 Search Strategy

Studies were identified through a title and abstract search of MEDLINE, Embase and Web of Science from 1/1/2000 to 15/7/2025. Each search combined relevant Medical Subject Headings and free words (including synonyms and abbreviations) for three concepts: Chronic kidney disease or kidney failure, conservative kidney management and kidney replacement therapy. The full search strategy is shown in File S2. Recent review articles and the reference lists of the final reports included in this analysis were screened to identify any additional studies.

Duplicate references were removed manually. Titles and abstracts were independently screened by two reviewers. KR screened all references. G.P., D.X., J.S. and K.A. acted as second screeners. Full text review of all selected abstracts was carried out by K.R., with independent review by the aforementioned authors. Discrepancies in selection at each stage were discussed until consensus was reached.

### 2.2 Selection of Studies

The inclusion criteria were as follows: Observational studies of adult populations with KF, which include both a group who have selected a non‐dialysis care pathway and a comparator group who are preparing for or receiving KRT. Case reports, reviews, commentary and conference abstracts were excluded. Other exclusion criteria were studies unavailable in English, studies focused on dialysis withdrawal or purely end‐of‐life care, studies of renal transplant recipients and studies on acute kidney injury. Studies in which the non‐dialysis group was ambiguously defined (i.e., it was not clear if participants had chosen non‐dialysis care or if they simply did not yet require dialysis) were also excluded. In cases where cohorts overlapped in two or more studies, the study with the largest sample size was selected. Studies were grouped according to country and region of origin.

### 2.3 Data Extraction and Variables

Data was extracted from the selected studies by KR and recorded in a standardised Excel spreadsheet. The data extraction tool was devised following an initial review of the included studies, in order to ensure that variables could be extracted in a uniform and efficient manner. It was not formally piloted. For each study, author, title, year, country of origin, study design, source population, primary outcomes, inclusion and exclusion criteria and sample size were recorded. In addition, relevant baseline characteristics of the participants were collected. These included age, sex, primary renal disease, presence of comorbidities, measures of comorbidity, measures of frailty or performance status, ethnicity, socioeconomic status, educational attainment, immigration status, home support, religion and language.

The outcome of interest was a choice to receive or receipt of non‐dialysis care, rather than preparing for or receiving KRT. For studies in which KRT populations were separated by modality, the groups were combined according to guidelines from the Cochrane handbook (Higgins et al. 2024). Participants who were undecided about their treatment choice were excluded.

Exposures of interest were clinical, demographic and socioeconomic characteristics of the participants. The included studies were reviewed in order to identify all potential exposures reported in the data. After this initial review and discussion between the authors, those factors with sufficient data to support a quantitative analysis (defined as an exposure reported in at least 30% of total studies or the relevant subgroup) were selected for inclusion in meta‐analysis. Less commonly reported factors were recorded and collated in a narrative synthesis.

It was anticipated that the exposures would be measured and reported using a variety of different methods or tools and therefore they needed to be recorded in a comparable way that allowed meta‐analysis. Table 1 shows a list of the variables collected and how they were defined for the purposes of the quantitative analysis. For continuous variables, where only median and interquartile range were reported, the mean and standard deviation were estimated using the formula proposed by Wan (2014).

**Table 1:** Definitions of exposures and data collection methods for those variables considered for quantitative analysis.

Variable	Type of variable	Definition	Notes on data collection method
Age	Continuous	Mean age and standard deviation in years	*Quantitative analysis not possible due to differing age cut offs in each study (means therefore not comparable)*
Sex	Binary	Male versus female	
Primary renal disease	Binary	Diabetic nephropathy versus any other cause of renal disease	
Comorbidities	Binary	Presence versus absence of comorbid condition	*Where available presence of diabetes mellitus, ischaemic heart disease, heart failure, cognitive impairment/dementia, stroke and cancer were recorded*
Charlson comorbidity index (CCI)	Continuous	Mean CCI and standard deviation	*Recorded in the instance where CCI (without age‐adjustment) was reported as a continuous variable*
Ethnicity	Binary	White versus non‐white ethnicity	*Recorded in studies from Western countries only*
Socioeconomic status	Binary	Socioeconomically deprived versus not deprived	*Socioeconomic deprivation defined as Index of Multiple Deprivations deciles 1 to 3 in UK studies OR 1* ^*st*^ *quartile of Welsh Index of Multiple deprivations OR 2 most deprived quintiles in Spanish studies OR lowest monthly income groups (South East Asia) OR receipt of social security payments (Hong Kong)*.
Educational attainment	Binary	Low educational attainment versus other groups	*Low educational attainment defined as illiteracy OR primary school education or less*
Living alone/single	Binary	Living alone versus living with other people/institutional living	*The definition of living alone included ‘social isolation’ or being ‘unsupported’ as described by two studies*

### 2.4 Risk of Bias Assessment

The Newcastle‐Ottawa Scale (NOS), adapted for this cross‐sectional comparative analysis of two groups, was used to assess study quality and risk of bias (Wells et al. n.d.). Whilst not validated for use in cross‐sectional analyses, there is precedent for use of a modified NOS in this context (Alshabanat et al. 2015; Modesti et al. 2016; Wiss and Brewerton 2020; Zhang et al. 2024). The adapted version used here is shown in File S3. Risk of bias assessment was carried out by KR. Studies were assessed according to the potential for selection bias, comparability of the study groups and measurement of the outcome. Studies which selected non‐dialysis care and KRT groups at the point that a treatment decision was made or compared a stage G4/5 CKD population choosing non‐dialysis care with an incident KRT group were considered sufficiently comparable. Groups were considered less comparable when patients on a non‐dialysis care pathway were compared to a prevalent dialysis population or when groups were selected using different methods or from different centres. A score out of 10 was assigned to each study. A score of 7 or above was considered high quality, a score of 4 to 6 was considered moderate quality and a score of 3 or less was considered low quality.

### 2.5 Analysis Approach

The characteristics of the included studies were summarised and a narrative synthesis of results was undertaken. It is not possible to draw comparisons between patients making treatment decisions in choice‐restricted versus shared decision making contexts (Hole et al. 2024). The analysis was, therefore, restricted to the latter.

Meta‐analysis was performed using Stata 17. Those exposures for which it was possible to combine data for meaningful comparison (i.e., the reported variables were judged to be measuring the same phenomenon and were reported in a sufficient number of studies) were selected for meta‐analysis. Heterogeneity between studies was assessed using a *Q* statistic. Where Q was large and *p* < 0.05, a random‐effects model was used. For binary variables, a summary odds ratio (OR) and 95% confidence interval (95% CI) were used to estimate the association of each variable with choosing non‐dialysis care, rather than KRT. For continuous variables, Hedges *g* and a 95% CI were used as a measure of association. Data were presented visually using forest plots.

Subgroup analysis was performed first by country or region (UK, mainland Europe, Australia, North America, East Asia, Southeast Asia). The UK operates under a single national health system (albeit with regional variation) and has generated a significant proportion of data in this area. It has, therefore, been considered separate to the rest of Europe. Secondly, based on possible sociocultural differences in decision‐making, studies were grouped as Western countries versus Asia. Heterogeneity between subgroups was assessed using a Q statistic for group difference and was considered significant when *p* < 0.05. Meta regression models were used to explore change in effect size by mean age in each study cohort.

Decision‐making is likely to change at lower levels of eGFR, as the urgency and certainty of KRT increases. In order to account for this, we performed a sensitivity analysis in which models were restricted to those studies which limited the sample to patients with eGFR < 15.

### 2.6 Publication Bias

Studies comparing groups of patients receiving non‐dialysis care with those who opt for KRT are designed to explore a broad spectrum of outcomes, with only a proportion aiming specifically to investigate treatment choice. It is, therefore, not possible to make an assessment of publication bias for the purposes of this review.

## 3 Results

**Figure 1:**
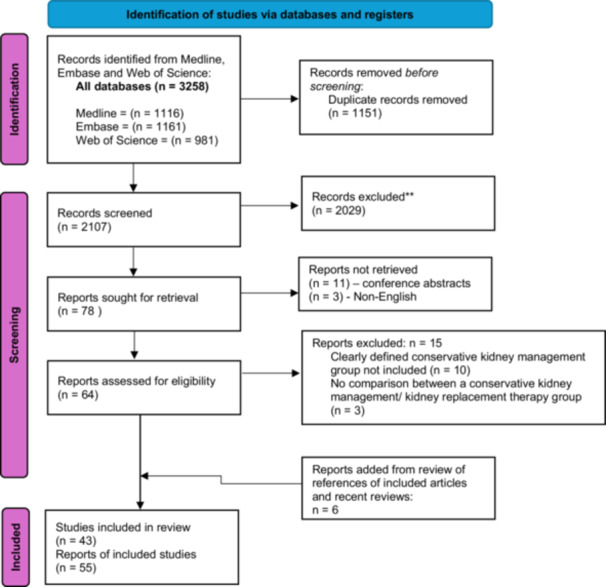
Flow diagram showing study selection procedure.

The study selection procedure is shown in Figure 1 (Page et al. 2021). A total of 43 studies, from 55 reports, encompassing 51,872 participants, were selected for final inclusion. Of these, 31 studies came from Western countries. These included four studies from Australia, 10 from the United Kingdom (UK), one from the UK and Australia, one from Canada, two from the United States (US) and 13 from mainland Europe. In addition, five studies came from Southeast Asia (Thailand, Malaysia and Singapore) and five from East Asia (all from Hong Kong). All of these are high‐ or middle‐income countries, in which KRT is theoretically available to all and treatment choices are made through a shared decision‐making process. These 41 studies encompassed 51 340 participants, 7402 receiving non‐dialysis care and 43,938 planning for or receiving KRT, and are considered in the analysis below. Finally, two studies came from Africa (Ghana and South Africa), both of which are settings in which access to KRT is limited and treatment decisions are choice‐restricted (Mathew et al. 2023; Okyere et al. 2022). Given the paucity of data from low resource settings, these studies were excluded from comparative analysis. The characteristics of all studies and references are summarised in Table 2.

**Table 2:** Characteristics of the included studies.

Source	Reference	Year	Country	Design	Prospective/retrospective	Single/multi‐centre	Source population	Age range (years)	eGFR^1^ (ml/min/1.73m^2^)	Primary outcomes	Sample size	Modified Newcastle‐Ottawa Score
*Studies from Australia, n = 4*												
Brown et al.	Brown et al. (2015)	2015	Australia	Cohort	Prospective	Single	Patients referred to a pre‐dialysis or Conservative Kidney Management (CKM) clinic; following shared decision‐making with a nephrologist	≥ 18	≤ 30	Survival time	395	8
Chou et al.	Chou et al. (2023)	2023	Australia	Cohort	Prospective	Single	Adult patients who started dialysis or attended the Conservative Kidney Management clinic for the first time	≥ 65	≤ 30	Survival time	510	8
Morton et al.	Morton et al. (2016, 2012)	2012; 2016	Australia	Cohort	Prospective	Multicentre	Incident patients with CKD (chronic kidney disease) 5 (including dialysis, pre‐emptive transplant recipients) and eGFR < 15 and planned for CKM presenting to their local renal unit	All	< 15 or dialysis/transplant	Treatment choice and education received; change in modality	721	10
So et al.	So et al. (2022)	2022	Australia	Cohort	Retrospective	Single	All patients referred to a Conservative Kidney Management clinic, with an available Quality of Life assessment. Sample includes all CKM patients and a selected group of KRT patients	≥ 18	< 15 or dialysis/transplant	Health related quality of life	604	5
*Studies from the United Kingdom, n = 11 (including 1 study which included a group of Australian patients)*												
Carson etc al.	Carson et al. (2009)	2009	United Kingdom	Cohort	Retrospective	Single	All new starters on dialysis and all patients referred to a pre‐dialysis clinic and who chose CKM	≥ 70	< 10.8 or dialysis	Survival time and hospitalisation	202	8
Chandna et al.	Chandna et al. (2011)	2011	United Kingdom	Cohort	Retrospective	Single	All patients with a GFR measurement between 10 and 15 and subsequent GFRs < 15	≥ 18	10 to 15	Survival time	844	8
Chanouzas et al.	Chanouzas et al. (2012)	2012	United Kingdom	Cross‐sectional	Prospective	Single	Incident pre‐dialysis patients, with a confirmed modality choice following an education programme	≥ 18	≤ 25	Treatment choice	118	7
Chess et al.	Chess et al. (2024)	2024	United Kingdom	Cohort	Retrospective	Multicentre	All adult patients who underwent pre‐dialysis education in Wales	≥ 18	No cut off	Survival time	1317	8
Hussain et al.	Hussain et al. (2013)	2013	United Kingdom	Cohort	Retrospective	Single	Patients referred to a pre‐dialysis service between 2006 and 2010 and who were offered both KRT and CKM options.	≥ 70	≤ 20	Survival time, risk factors for mortality and palliative care referral	441	8
Pyart et al.	Pyart et al. (2020)	2020	United Kingdom	Cohort	Retrospective	Single	All patients referred for pre‐dialysis education	≥ 70	≤ 20	Treatment choice and survival time	1216	9
Raman et al.	Raman et al. (2018)	2018	United Kingdom	Cohort	Prospective	Single	Patients enroled in the Salford Kidney study once age > 75 and GFR < 15 and who subsequently chose a treatment modality	≥ 75	≤ 15	Survival time, cause of death, hospitalisation	204	7
Rosenberg et al.	Rosenberg et al. (2023)	2023	United Kingdom	Cohort	Retrospective	Single	All new referrals to a pre‐dialysis clinic, who subsequently chose CKM or started dialysis	≥ 65	≤ 30	Treatment choice, hospitalisation	824	8
Murtagh et al.	Murtagh et al. (2007)	2007	United Kingdom	Cohort	Retrospective	Multicentre	All patients receiving pre‐dialysis care and with a confirmed decision to have either dialysis or CKM	≥ 70	No cut off	Survival time	129	8
Shah et al.	Shah et al. (2019)	2019	United Kingdom/Australia	Cross‐sectional	Prospective	Multicentre	All patients > 75 years old on dialysis or with eGFR < 10 and receiving CKM	≥ 75	< 10 or dialysis	Health‐related quality of life	129	6
O'Keefe et al.	O'Keeffe et al. (2025)	2025	United Kingdom	Cohort	Retrospective	Single	All patients referred to a pre‐dialysis clinic between 2011 and 2018	≥ 18	≤ 20	Treatment choice and survival time	1957	8
*Studies from North America, n = 3*												
Scherer et al.	Scherer et al. (2023)	2023	United States of America	Cross‐sectional	Prospective	Multicentre	Adult patients with chronic kidney disease, enroled in CKDOPPS study	≥ 18	≤ 30	Treatment choice and modalities discussed with patient	1018	9
Wong et al.	Wong et al. (2018, 2016)	2016; 2018	United States of America	Cohort	Retrospective	Multicentre	Patients in the VA database with a sustained GFR < 15 and those with a dialysis procedure code/USRDS registration after cohort entry.	≥ 18	≤ 15	Treatment choice; palliative care referral	21093	8
Tam‐Tham et al.	Tam‐Tham et al. (2020, 2018)	2018; 2020	Canada	Cohort	Retrospective	Multicentre	Adult patients in Alberta with 2 x eGFR values < 10 (at least 90 days apart)	≥ 65	≤ 10	Survival time; hospitalisation and intensity of care	968	7
*Studies from Europe, n = 13*												
Reindl‐Schwaighofer et al.	Reindl‐Schwaighofer et al. (2017)	2017	Austria	Cohort	Retrospective	Multicentre	Incident haemodialysis patients (from a national register) and conservatively managed patients, with eGFR < 10, in a single centre in the same time period	≥ 65	≤ 10 or dialysis	Survival time	8796	6
Moranne et al.	Moranne et al. (2018)	2018	France	Cohort	Prospective	Multicentre	Patients with non‐dialytic CKD with at least 1 previous nephrology clinic appointment	≥ 75	≤ 20	Survival time and dialysis initiation	339	8
Joly et al.	Joly et al. (2003)	2003	France	Cohort	Retrospective	Single	Incident pre‐dialysis patients, seen within one renal unit	≥ 80	CrCl < 10	Survival time and treatment choice	144	8
Martino et al.	Martino et al. (2024)	2024	Italy	Case‐control	Retrospective	Single	Incident patients reaching ESKD and commencing HD or enroling in an CKM programme and completing 6 months of chosen treatment	≥ 75	≤ 12 or dialysis	Health‐related quality of life	50	6
van Loon et al	Goto et al. (2019); van Loon et al. (2019)	2018; 2019	the Netherlands	Cohort	Prospective	Multicentre	Dialysis patients within 3 weeks prior or 2 weeks post start of maintenance dialysis and CKM patients once eGFR < 15	≥ 65	≤ 15 or dialysis	Prevalence of geriatric impairments and frailty; health‐related quality of life	285	9
Verberne et al.	Verberne et al. (2016, 2018, 2020)	2016; 2018; 2020	the Netherlands	Cohort	Retrospective	Single	All patients seen in an advanced CKD clinic and who had made a decision to plan for dialysis or CKM	≥ 70	≤ 20	Survival time, quality of life and treatment burden; healthcare cost	366	8
Arenas et al.	Arenas et al. (2024)	2024	Spain	Cross‐sectional	Retrospective	Single	All patients referred to an advanced CKD clinic for treatment decision‐making	≥ 18	≤ 30	Treatment choice	673	7
Garcia et al.	García Testal et al. (2021)	2020	Spain	Cohort	Retrospective	Single	Patients seen in an advanced CKD clinic, who subsequently chose CKM or started dialysis	≥ 80	≤ 15 or dialysis	Survival time and healthcare usage	87	7
Guerrero Riscos et al.	Guerrero Riscos et al. (2019)	2019	Spain	Cohort	Prospective	Multicentre	Incident patients in an advanced CKD, who received pre‐dialysis education	≥ 18	≤ 30	Survival time and hospital admissions	166	9
Villareal et al.	Rodriguez Villarreal et al. (2014)	2014	Spain	Cohort	Prospective	Single	Patients in pre‐dialysis clinic	≥ 75	≤ 30	Treatment choice and survival time	56	7
Teruel et al.	Teruel et al. (2015)	2015	Spain	Cohort	Retrospective	Single	Patients with eGFR < 15 seen within the nephrology service	≥ 18	≤ 15	Treatment choice and survival time	232	9
Guitierrez Sanchez et al.	Gutiérrez Sánchez et al. (2019)	2017	Spain	Cross‐sectional	Prospective	Single	Patients with eGFR < 15 receiving dialysis or on a CKM pathway	≥ 18	≤ 15	Symptom burden	123	5
Garcia Garcia et al.	García García et al. (2007)	2007	Spain	Cohort	Retrospective	Single	Adult patients who started dialysis or chose CKM between 1992 to 1995 and 2000 to 2003	≥ 18	≤ 15	Treatment choice and survival time	420	5
*Studies from Hong Kong, n = 5*												
Chan et al.	Chan et al. (2010)	2010	Hong Kong	Cohort	Retrospective	Single	Adult patients referred for dialysis assessment	≥ 18	≤ 15 or ≤ 10 with diabetes	Treatment choice, resuscitation status and symptom burden	213	8
Kwok et al.	Kwok et al. (2016)	2016	Hong Kong	Cohort	Retrospective	Single	Incident patients referred for renal advanced care planning	≥ 65	≤ 15	Survival time	558	8
Shum et al.	Shum et al. (2014)	2014	Hong Kong	Cohort	Retrospective	Single	All patients receiving dialysis assessment	≥ 65	≤ 15	Survival time and hospitalisation	199	8
Yong et al.	Yong et al. (2009)	2009	Hong Kong	Cross‐sectional	Prospective	Single	Prevalent patients on PD or HD for at 3 months and renal palliative care patients with CrCl < 15	≥ 18	CrCl ≤ 15 or dialysis	Health related quality of life and symptom burden	179	7
Tse et al. and Yuen et al.	Tse (2009); Yuen et al. (2016)	2009; 2016	Hong Kong	Cohort	Retrospective	Single	Incident patients referred for renal advanced care planning and dialysis education	≥ 18	Cr > 450 or > 350 with diabetes	Treatment choice and survival time	600	8
Studies from South East Asia, *n* = 5												
Wan et al.	Wan Zukiman et al. (2017)	2017	Malaysia	Cross‐sectional	Prospective	Multicentre	Patients with CKD 5, either on dialysis for at least 1 year or those who had attended pre‐dialysis education and decided not to have dialysis	≥ 18	≤ 15 or dialysis	Symptom burden, anxiety and depression	187	7
Ch'ng et al.	Ch'ng et al. (2020)	2020	Malaysia	Cross‐sectional	Retrospective	Multicentre	Incident, adult patients with eGFR < 10 and deemed to require KRT	≥ 18	≤ 10	Survival time	3990	5
Seow et al.	Seow et al. (2013)	2013	Singapore	Cohort	Prospective	Single	Patients with pre‐dialysis CKD and either age adjusted Charlson Comorbidity Index > 8 or age > 75	≥ 75	8 to 12	Health‐related quality of life	101	7
Teo et al.	Teo et al. (2010)	2010	Singapore	Cohort	Retrospective	Single	Adult patients, diagnosed with ESKD and survived > 90 days	≥ 18	≤ 15	Survival time, hospitilisation, treatment choice	168	6
Noppakun et al.	Noppakun et al. (2024)	2024	Thailand	Cohort	Retrospective	Single	All new starters on dialysis and those with eGFR < 15 who chose CKM	≥ 70	≤ 15 or dialysis	Survival time	719	8
*Studies from Africa, n = 2*												
Mathew et al.	Mathew et al. (2023)	2023	South Africa	Cross‐sectional	Prospective	Single	Convenience sample of patients on dialysis or CKM	≥ 18	≤ 20 or dialysis	Health‐related quality of life, anxiety and depression	150	6
Okyere et al.	Okyere et al. (2022)	2022	Ghana	Cohort	Retrospective	Single	Patients reaching end stage kidney disease (i.e., eGFR < 15 or started dialysis)	≥ 10	≤ 15 or dialysis	Treatment choice	382	7

### 3.1 Risk of Bias

All included studies were of moderate or high quality, with NOS scores ranging from 5 to 10 (File S4). A number of studies were limited by missing data and this particularly affected socioeconomic variables. In addition, non‐response rate was poorly reported amongst prospective studies. Sampling methods, however, were well‐described and the majority of studies included a sample representative of the target population. The non‐dialysis care pathway and KRT groups were sufficiently comparable in most studies.

### 3.2 Clinical Factors

Older age was associated with receiving non‐dialysis care in all studies. This association was not quantified as age cut‐offs varied between studies and, therefore, mean ages were not comparable.

**Figure 2:**
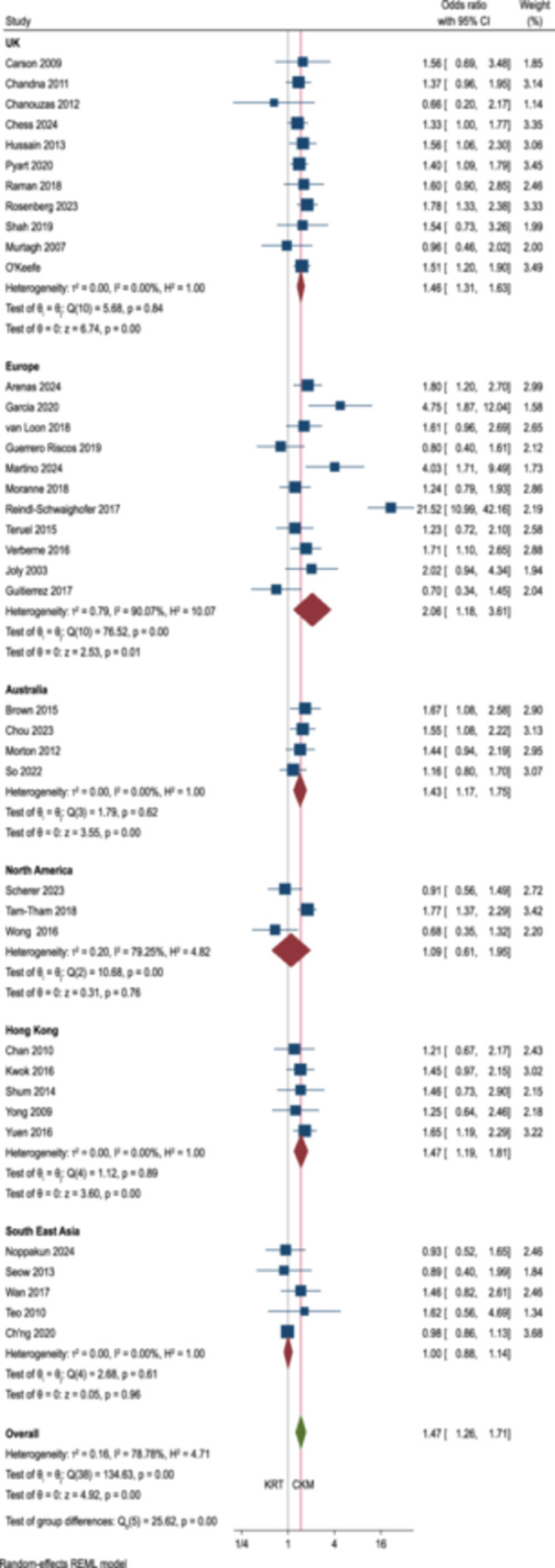
Random effects model for the association of female sex with choosing conservative kidney management by country/region.

Female sex (reported in 39 studies) was associated with receipt of non‐dialysis care rather than KRT overall, with a pooled OR of 1.47 (95% CI 1.26–1.71). Two studies did not report sex(García García et al. 2007; Rodriguez Villarreal et al. 2014). This association was demonstrated in all Western regions, with the exception of North America. Of the three North American studies, one from the US (Wong et al. 2018, 2016)included only nine female patients (out of 21 093) (Figure 2). Female sex was associated with non‐dialysis care in Hong Kong (OR 1.47, 95% CI 1.19 to 1.82), but not in South East Asia. When subgroup analysis was performed by Western countries versus Asia, female sex was associated with non‐dialysis care in both groups (OR 1.55, 95% CI 1.26–1.91 and OR 1.23, 95% CI 1.01–1.51, respectively). In a meta regression model, the effect size increased as mean age of the study cohort increased (File 6).

**Figure 3:**
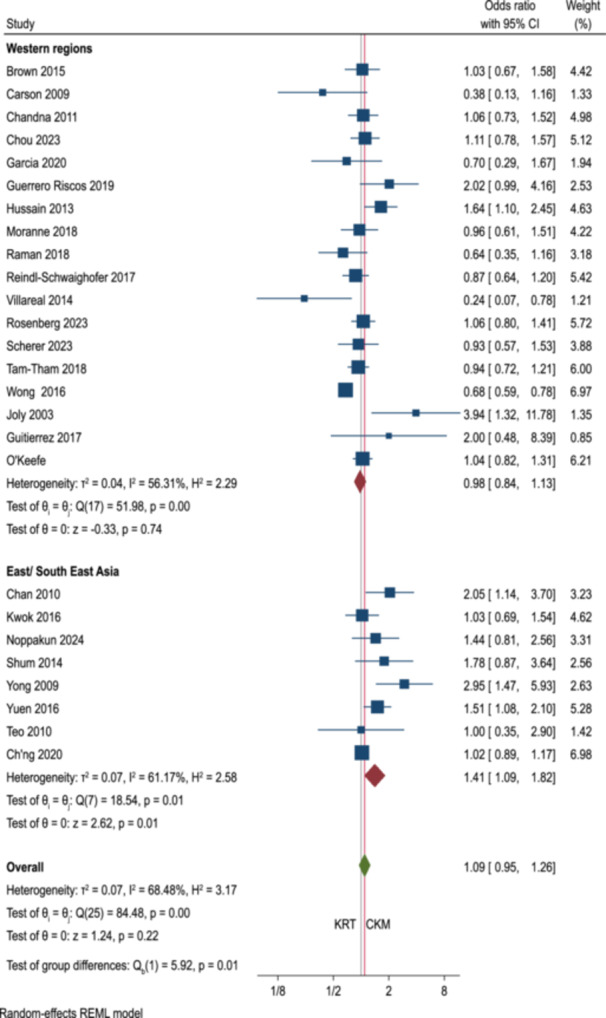
Random effects model for the association of diabetes mellitus with choosing conservative kidney management in Western regions and Asia.

As shown in Figure 3, comorbid diabetes (reported in 26 studies) was not associated with treatment pathway overall (pooled OR 1.09, 95% CI 0.95–1.26). In a subgroup analysis, diabetes was associated with having non‐dialysis care in Asia but not Western regions (OR 1.41, 95% CI 1.09–1.82). Cognitive impairment or dementia was associated with having non‐dialysis care in 11 studies out of the 14 in which it was reported. Cognitive impairment, dementia or an inability to provide informed consent was an exclusion criteria in seven studies (Goto et al. 2019; Gutiérrez Sánchez et al. 2019; Noppakun et al. 2024; Raman et al. 2018; Shah et al. 2019; van Loon et al. 2019; Wan Zukiman et al. 2017; Yong et al. 2009). History of stroke was reported in 14 studies and associated with treatment choice in 8 of these. Heart failure was reported in 16 studies and associated with receiving non‐dialysis care in 10.

**Figure 4:**
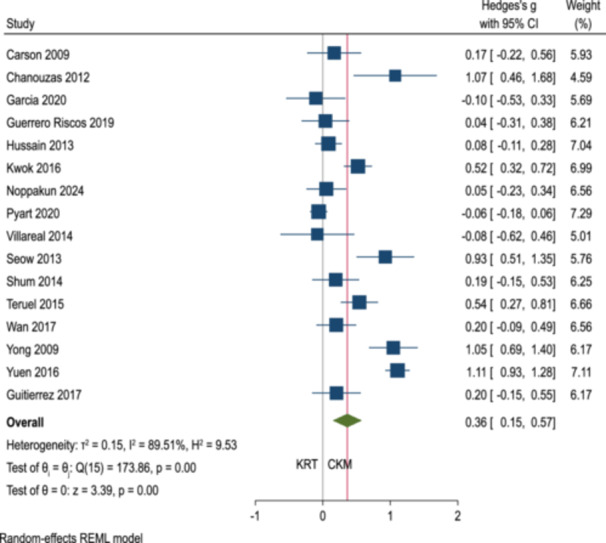
Random effects model for the association of higher mean Charlson comorbidity index with choosing conservative kidney management.

Mean Charlson Comorbidity Index (CCI) (Charlson et al. 1987) was reported in 16 studies. Higher mean CCI was associated with receiving non‐dialysis care overall, as shown in Figure 4 (pooled Hedges g 0.36, 95% CI 0.15–0.57). There were no significant differences between subgroups.

Frailty or performance status were reported using a range of measures, which were not directly comparable to one another, and this exposure was excluded from meta‐analysis. Increased frailty or reduced performance status was associated with receipt of non‐dialysis care in 15 studies, with no difference between the groups demonstrated in a further four in which it was reported. Three of the studies reporting no difference were from the UK and Australia (Chou et al. 2023; Raman et al. 2018; So et al. 2023) and all reported Karnofsky Performance Status (KPS) (Mor et al. 1984). Patients with a KPS lower than 60 were excluded from one of these studies (Raman et al. 2018). Another compared a confirmed CKM group to a prevalent population of dialysis patients referred to a Kidney Supportive Care clinic for symptom management (So et al. 2023). This selected group may have been more frail than the average dialysis patient. The final study was from Hong Kong and reported on a fit population, in which 90% of participants in both groups were independently mobile (Shum et al. 2014).

### 3.3 Socioeconomic Factors

**Figure 5:**
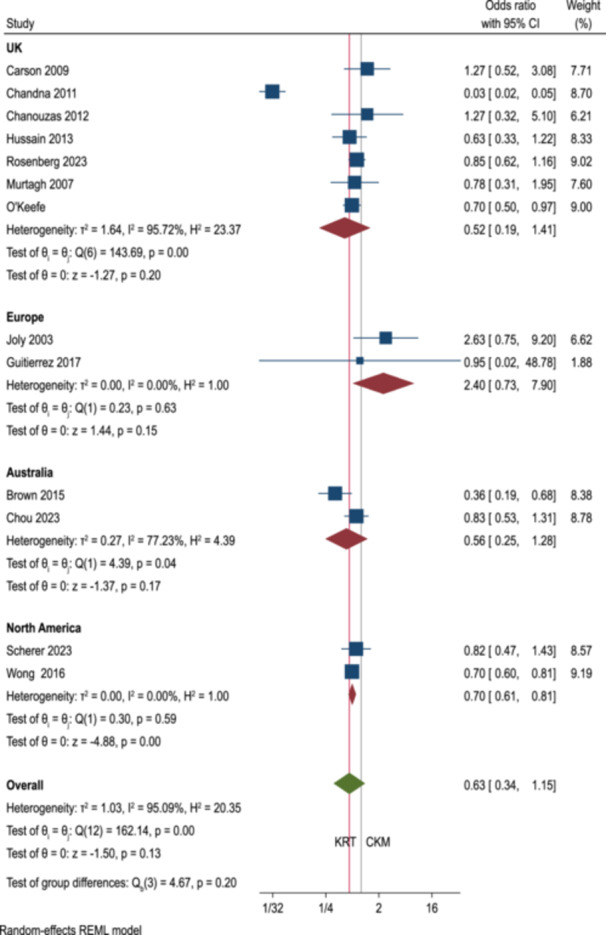
Random effects model for the association of non‐white ethnicity with choosing conservative kidney management in Western regions.

Ethnicity data was collected only from Western countries and was reported in 13 out of 31 studies (Figure 5). There was no overall association between non‐white ethnicity and treatment received (pooled OR 0.63, 95% CI 0.34–1.15). Non‐white ethnicity was associated with lower uptake of non‐dialysis care in North America only; in a group including two large studies from the US (OR 0.71, 95% CI 0.61–0.81) (Scherer et al. 2023; Wong et al. 2018, 2016).

Language (defined as presence of a language barrier or requiring an interpreter) was reported in three studies (UK, Australia and Spain) and no association with treatment choice was shown (Arenas et al. 2024; Morton et al. 2016, 2012; Rosenberg et al. 2023). Inability to communicate in native language was an exclusion criterium in five studies. Immigration status was reported in two studies; one from Australia which showed no association with choosing CKM (So et al. 2023, 2022) and one from Spain, which showed higher uptake of CKM amongst those with Spanish nationality compared to other groups(Arenas et al. 2024). Religion was reported in two studies and had no association with treatment choice (Rosenberg et al. 2023; Yong et al. 2009).

A measure of socioeconomic status was reported in 11 out of 41 studies. This number did not reach the threshold of 30% required for the variable to be included in meta‐analysis and methods of measuring socioeconomic status were highly heterogenous. The model comparing treatment choices between the most derived groups and the rest of participants is, therefore, included in the supplements (Supplementary file 5). Socioeconomic status was reported in five studies from Western countries (Australia, UK and Spain). All of these used area‐based measures of deprivation (derived from the participants' address) and no association with treatment choice was demonstrated (Arenas et al. 2024; Morton et al. 2012; O'Keeffe et al. 2025; Pyart et al. 2020; Rosenberg et al. 2023). In contrast, individual‐level measures of deprivation (i.e. receipt of a social support grant or monthly income) were reported in six Asian studies; four from Hong Kong (Kwok et al. 2016; Shum et al. 2014; Yong et al. 2009; Yuen et al. 2016) and two from Malaysia (Ch'ng et al. 2020; Wan Zukiman et al. 2017). Three of these studies demonstrated that uptake of non‐dialysis care was higher amongst the most deprived groups compared to others (Ch'ng et al. 2020; Kwok et al. 2016; Shum et al. 2014).

**Figure 6:**
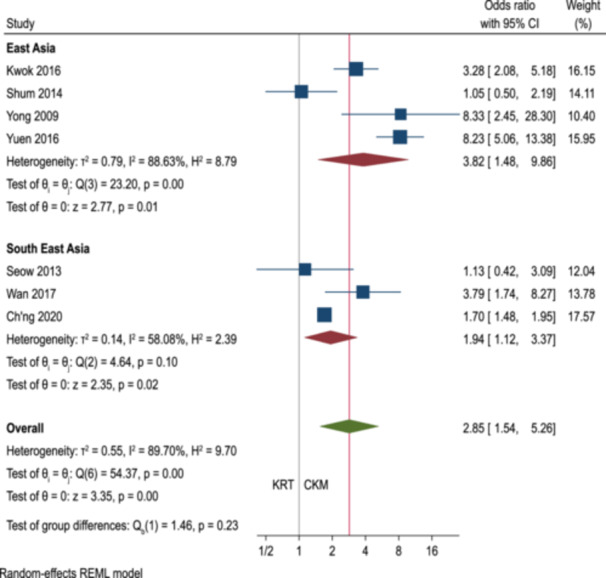
Random effects model for the association of low educational attainment with choosing conservative kidney management in East and Southeast Asia.

Other surrogates for low socioeconomic status were also associated with receiving non‐dialysis care in Asian countries. Educational attainment was reported in seven of the ten Asian studies (subgroup analysis shown in Figure 6), and low educational attainment (primary school or less or illiteracy) was associated with choosing non‐dialysis care (pooled OR 2.85, 95% CI 1.54–5.27). In addition, three Hong Kong studies reported an association between living in public housing and choosing non‐dialysis care rather than KRT (Kwok et al. 2016; Shum et al. 2014; Yuen et al. 2016). Socioeconomic surrogates were minimally reported in Western studies and the amount of missing data was significant. Education was reported in two studies in Australia and the UK, with no association demonstrated (Chanouzas et al. 2012; So et al. 2023, 2022). One Australian study reported that having private health insurance was associated with lower odds of choosing CKM (Morton et al. 2016, 2012).

**Figure 7:**
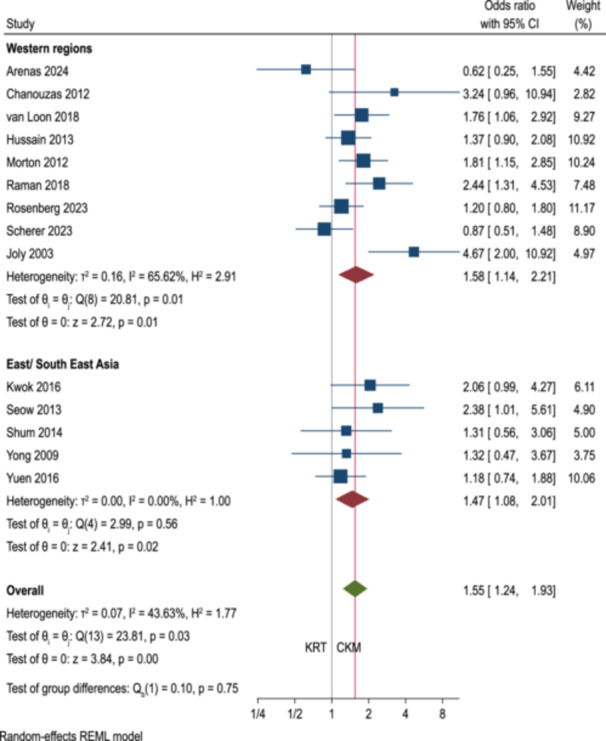
Random effects model for the association of living alone with choosing conservative kidney management in Western countries and Asia.

Levels of home or social support were reported in 14 studies. Living alone was associated with a receiving non‐dialysis care rather than KRT (Figure 7), with a pooled OR of 1.55 (95% CI 1.24–1.93). There were no significant differences between subgroups. Institutional living (i.e., residential or nursing home) was reported in eight studies and associated with choosing non‐dialysis care in four (Hussain et al. 2013; Kwok et al. 2016; Moranne et al. 2018; Tam‐Tham et al. 2020, 2018). Meta regression showed no significant change in effect size with mean age in each study (File S7).

### 3.4 Sensitivity Analysis

Out of 41 studies included in the meta‐analysis, 26 limited their samples to patients with an eGFR < 15 (Table 2). In a sensitivity analysis restricted to these studies in low eGFR groups, the associations of female sex, diabetes mellitus and mean CCI with treatment choice (including subgroup differences) were unchanged. Ethnicity was reported in only five Western studies in low eGFR populations, and therefore, this variable was excluded from the restricted analysis. All seven Asian studies included in the prior model for low educational attainment were in low eGFR groups. In a model restricted to nine studies in low eGFR populations (Joly et al. 2003; Kwok et al. 2016; Morton et al. 2012; Raman et al. 2018; Seow et al. 2013; Shum et al. 2014; van Loon et al. 2019; Yong et al. 2009; Yuen et al. 2016), living alone was again associated with choosing non‐dialysis care (pooled OR 1.83, 95% CI 1.43–2.33). This association was demonstrated in both Western countries and Asia.

### 3.5 Multivariable Analysis

Only seven studies out of the 41 included in the meta‐analysis reported adjusted odds ratios for the association of exposures of interest with treatment choice (Morton et al. 2012; Pyart et al. 2020; Rodriguez Villarreal et al. 2014; Rosenberg et al. 2023; Scherer et al. 2023; Teruel et al. 2015; Wong et al. 2016). The models were highly heterogenous (in terms of the variables included) and therefore non‐comparable. As a result, no multivariable analysis was possible, and we were unable to examine the impact of confounding factors on the observed associations.

## 4 Discussion

In this systematic review we report that female sex, frailty, older age and higher burden of comorbidities are associated with choosing non‐dialysis care, rather than KRT. There was minimal geographical variation in the clinical predictors of treatment choice. Socioeconomic factors, however, demonstrated some regional differences. Non‐white ethnicity was associated with reduced uptake of non‐dialysis care in the US only. Socioeconomic deprivation and low educational attainment were associated with non‐dialysis care in Asia; whilst area‐level measures of socioeconomic status did not predict treatment choice in Western countries. Living alone, however, was associated with choosing non‐dialysis care overall and in both Western regions and Asia.

It is intuitive that older age, frailty and higher comorbidity burden are associated with receipt of non‐dialysis care. Female sex has been associated with increased uptake of palliative care across a range of health conditions (Rashid et al. 2022), as well as higher likelihood of dialysis withdrawal (Leggat et al. 1997). This may suggest that women are less likely to choose intensive treatments towards end of life and, therefore, are more likely to opt for a non‐dialysis care pathway. The underlying reasons for this are not understood and require further exploration. It is likely that both sociocultural factors, related to treatment preference, and barriers to KRT access are at play. It has been suggested that uptake of KRT is reduced amongst older women as they are more likely to survive a male partner and live alone in later years(Antlanger et al. 2019). Our analysis shows that living alone or being widowed are associated with increased uptake of non‐dialysis care, therefore, this may be part of the explanation. Our meta regression analysis, showing that the effect size for this association increased in older cohorts, also supports this hypothesis. In addition, one qualitative study suggested that women tended to engage with treatment decision‐making and seek information earlier in the process; whilst men struggled more with acceptance and tended to delay. It is possible that if men are faced with more urgent decisions, at later and more symptomatic stages of disease, they are more likely to default to KRT (Harwood and Clark 2014).

Non‐white ethnicity was associated with reduced uptake of non‐dialysis care in the US only. This is consistent with American data which suggests that people from ethnic minority backgrounds are less likely to access palliative care (Foley et al. 2018; Rashid et al. 2022; Wen et al. 2019) or engage in advanced care planning (Eneanya et al. 2016). Previous qualitative studies have suggested that reduced access to CKM in non‐white groups in the US may be due to systemic or clinician related‐factors (Ladin et al. 2021), as well as spiritual and cultural beliefs and institutional mistrust amongst ethnic minority groups(Carr 2011; Kurella Tamura et al. 2010). Black and Latino patients report ‘interpersonal and structural’ challenges to treatment decision‐making (Vélez‐Bermúdez et al. 2022), which may create barriers to nuanced discussions around KRT and non‐dialysis options. The reasons for the observed association are likely multifactorial. Our univariate analysis does not account for other sociodemographic factors, such as health insurance status, socioeconomic deprivation or educational attainment, all of which are determinants of healthcare access and were not reported in the US. Nevertheless, our findings highlight the importance of social, political and cultural context in treatment decision‐making. Whilst no association between ethnicity and uptake of non‐dialysis care was seen in the UK, Europe or Australia, it was under‐reported as an exposure (particularly in Europe) and data was affected by missingness. This highlights a crucial evidence gap, particularly given the increasing cultural diversity and growing populations of older people from immigrant backgrounds in most European countries(Van Mol et al. 2016).

Socioeconomic deprivation was associated with increased uptake of non‐dialysis care in Asia. This association may be more indicative of barriers to accessing KRT than a difference in treatment preference; albeit we were unable to adjust for clinical factors, such as comorbidities, which are likely to influence treatment decisions and may differ between socioeconomic groups. This finding may demonstrate that, even within settings where KRT is theoretically available to all, socioeconomic impediments to access still exist. Lower socioeconomic groups may find the cost of travel for dialysis prohibitive or have limited support due to caregivers need to work. In the case of Hong Kong, a ‘peritoneal dialysis first’ policy is employed. Those living in more crowded spaces or without a fixed address may not have sufficient storage room for dialysis fluid and groups with lower health literacy may lack confidence to take on a home modality. Previous studies examining access to healthcare in deprived, East Asian communities have reported perceived stigma and challenges navigating the public system as barriers(Tan et al. 2023). These factors may inhibit patients from engaging in complex, chronic treatments such as KRT. Socioeconomic status was reported in only five of 30 Western studies and all used area‐level measures, which may lack granularity. Given the paucity of data, it is difficult to draw inferences about the impact of socioeconomic status on uptake of non‐dialysis care pathways. Once again, this represents an important evidence gap and further studies, reporting individual measures of deprivation, are required.

Finally, living alone or being socially isolated was associated with increased uptake of non‐dialysis care. Qualitative data suggests that families (particularly spouses) are closely involved with treatment decision‐making and may influence patients to opt for treatments perceived to prolong life(Dahm et al. 2024; Han et al. 2019; Kimmitt et al. 2025). In addition, evidence suggests that patients may be more willing to face burdensome treatments if they believe it will allow them to spend more time with family. Those without family may feel more comfortable choosing an option which prioritises quality of life over quantity (Harwood and Clark 2014; Hole et al. 2025; Kimmitt et al. 2025). Whilst this finding illustrates the interpersonal complexities of treatment decision‐making, it may also highlight additional barriers to KRT access. Older people who are socially isolated may feel less physically and emotionally equipped to manage the burden of dialysis, may struggle with travel to a dialysis unit or be unable to take on a home modality (such as peritoneal dialysis) without support. This finding highlights the growing role for assisted peritoneal dialysis in an aging KF population; a treatment option which is ideally suited to more frail people who would benefit from KRT, but is not ubiquitous globally or within the UK (Brown et al. 2022).

The strengths of this review are that it combines and compares evidence from 13 countries across Asia, Europe, Australia and North America and includes a large pooled sample size. It is also, to our knowledge, the first quantitative synthesis of data aiming to identify predictors of choosing a non‐dialysis care pathway.

There are, however, some important limitations. Multivariable analysis was not possible and this means that we were unable to explore the impact of confounding factors on the observed associations. Additionally, quality was only rated by one assessor which may limit its validity. We excluded studies written in languages other than English, so although we included studies from a range of countries, we may have missed studies from some countries, limiting the generalisability of this work. Whilst clinical exposures were consistently reported, socioeconomic variables were notably under‐reported and suffered from missing data. In particular, data on religion, language barrier, immigration status, health insurance and educational attainment were poorly reported. Socioeconomic data was reported in a range of different ways, which was not always possible to combine for quantitative comparison, and this limited the scope of the meta‐analysis. Whilst these factors may limit the robustness of our conclusions, they also highlight essential gaps in knowledge and constitute important findings in themselves.

## 5 Implications for Practice

As discussed above, our findings highlight the increasing importance of assisted PD programmes as the KF population ages and becomes more frail. Additionally, an important challenge in this data synthesis was significant heterogeneity in the inclusion criteria for non‐dialysis populations and how they were identified in each study. Some, otherwise high‐quality, studies had to be excluded as the non‐dialysis groups were too ambiguously defined. Unlike those who opt for KRT, patients receiving non‐dialysis care are often not captured in disease registers (Stel et al. 2021). This highlights a challenge for researchers, clinicians and policy‐makers as this group is more challenging to identify and describe. Establishing detailed registries of patients receiving non‐dialysis care is, therefore, a priority and would aid in assessing the unique needs of this population.

Finally, as the KF population ages and becomes more medically complex, it is increasingly important to recognise and understand possible barriers to appropriate treatment decision‐making. Improving our knowledge of where challenges arise may help to identify under‐served groups, inform the implementation of CKM and KRT programmes and aid holistic conversations around goals of care.

## 6 Conclusions

The findings of this review have illustrated the complexity of treatment decision‐making in KF. It is not possible from this analysis to understand whether the observed associations arise from appropriate shared decisions, challenges to the decision‐making process or barriers to KRT access. Nevertheless, this review lays the foundation for future research focused on interrogating these associations. Furthering our understanding will require studies capturing robust and detailed socioeconomic data and qualitative work exploring the patient perspective. High quality research would be aided by the inclusion of patients receiving non‐dialysis care in disease registers. Finally, we demonstrate global variation in predictors of uptake of non‐dialysis care pathways, indicate the importance of context‐specific data, and highlight an essential gap in evidence from low‐resource and choice restricted settings.

## Author Contributions

K.R. was responsible for study design, literature search, study selection, data collection and analysis, as well as writing the first draft of the manuscript. G.P., J.S., D.X. and K.A. performed independent screening of titles, abstracts and full texts. B.C. was involved in developing the research question, study design and editing of the manuscript. N.M. and A.B. provided mentorship and contributed to the development of the final manuscript.

## Funding

The authors have nothing to report.

## Conflicts of Interest

The authors declare no conflicts of interest.

## Supporting information

Supporting File 1

Supporting File 2

## Data Availability

The data underlying this article are available in the article and in its online supplementary material. The template data collection forms are available from the corresponding author on request.
