# Supplementary material for: Global Variation in Predictors of Uptake of Conservative Kidney Management: A Systematic Review and Meta‐Analysis
Source: J Ren Care. 2026 Jul 4;52(3):e70071. doi: 10.1111/jorc.70071 (PMC13332701; doi:10.1111/jorc.70071)
Supplement: Supplementary file 2 — Supporting File 2 [file JORC-52-0-s002.docx]

**Supplementary file 2: Full search strategy for both MEDLINE, Embase and Web of Science**

**Ovid MEDLINE:**

| 1. | exp Renal Insufficiency, Chronic/ |
| --- | --- |
| 2. | exp Kidney Failure, Chronic/ |
| 3. | chronic kidney disease.tw. |
| 4. | ckd.tw. |
| 5. | end-stage kidney disease.tw. |
| 6. | eskd.tw. |
| 7. | end stage renal failure.tw. |
| 8. | esrf.tw. |
| 9. | advanced kidney disease.tw. |
| 10. | kidney disease.tw. |
| 11. | end stage renal disease.tw. |
| 12. | esrd.tw. |
| 13. | kidney failure.tw. |
| 14. | renal failure.tw. |
| 15. | 1 or 2 or 3 or 4 or 5 or 6 or 7 or 8 or 9 or 10 or 11 or 12 or 13 or 14 |
| 16. | *Conservative Treatment/ |
| 17. | exp Palliative Care/ |
| 18. | renal supportive care.tw. |
| 19. | kidney supportive.tw. |
| 20. | supportive care.tw. |
| 21. | (conservative adj2 management).tw. |
| 22. | palliative.tw. |
| 23. | non-dialytic.tw. |
| 24. | nondialytic.tw. |
| 25. | *Palliative Medicine/ |
| 26. | (conservative adj2 care).tw. |
| 27. | 16 or 17 or 18 or 19 or 20 or 21 or 22 or 23 or 24 or 25 or 26 |
| 28. | exp Renal Dialysis/ |
| 29. | *Renal Replacement Therapy/ |
| 30. | dialysis.tw. |
| 31. | haemodialysis.tw. |
| 32. | hemodialysis.tw. |
| 33. | peritoneal dialysis.tw. |
| 34. | HD.tw. |
| 35. | PD.tw. |
| 36. | kidney replacement therapy.tw. |
| 37. | renal replacement therapy.tw. |
| 38. | krt.tw. |
| 39. | rrt.tw. |
| 40. | 28 or 29 or 30 or 31 or 32 or 33 or 34 or 35 or 36 or 37 or 38 or 39 |
| 41. | 15 and 27 and 40 |

**Ovid Embase**

| 1. | exp chronic kidney failure/ |
| --- | --- |
| 2. | chronic kidney disease.tw. |
| 3. | ckd.tw. |
| 4. | kidney disease.tw. |
| 5. | advanced kidney disease.tw. |
| 6. | end stage renal failure.tw. |
| 7. | esrf.tw. |
| 8. | end stage renal disease.tw. |
| 9. | esrd.tw. |
| 10. | end stage kidney disease.tw. |
| 11. | eskd.tw. |
| 12. | kidney failure.tw. |
| 13. | renal failure.tw. |
| 14. | 1 or 2 or 3 or 4 or 5 or 6 or 7 or 8 or 9 or 10 or 11 or 12 or 13 |
| 15. | *palliative therapy/ |
| 16. | *conservative treatment/ |
| 17. | palliative.tw. |
| 18. | renal supportive care.tw. |
| 19. | kidney supportive care.tw. |
| 20. | supportive care.tw. |
| 21. | (conservative adj2 care).tw. |
| 22. | (conservative adj2 management).tw. |
| 23. | non-dialytic.tw. |
| 24. | nondialytic.tw. |
| 25. | 15 or 16 or 17 or 18 or 19 or 20 or 21 or 22 or 23 or 24 |
| 26. | exp hemodialysis/ |
| 27. | exp peritoneal dialysis/ |
| 28. | exp renal replacement therapy/ |
| 29. | kidney replacement therapy.tw. |
| 30. | renal replacement therapy.tw. |
| 31. | krt.tw. |
| 32. | rrt.tw. |
| 33. | dialysis.tw. |
| 34. | haemodialysis.tw. |
| 35. | hemodialysis.tw. |
| 36. | peritoneal dialysis.tw. |
| 37. | HD.tw. |
| 38. | PD.tw. |
| 39. | 26 or 27 or 28 or 29 or 30 or 31 or 32 or 33 or 34 or 35 or 36 or 37 or 38 |
| 40. | 14 and 25 and 39 |

**Web of Science**

**chronic kidney disease** (Topic) or **ckd** (All Fields) or **end stage kidney disease** (All Fields) or **eskd** (All Fields) or **end stage renal disease** (All Fields) or **esrd** (All Fields) or **end stage renal failure** (All Fields) or **esrf** (All Fields) or **kidney failure** (All Fields) or **renal failure** (All Fields)

**And**

**conservative kidney management** (Topic) and **Conservative Care** (OR – Search within topic) and **Kidney Supportive Care** (OR – Search within topic) and **Renal Supportive Care** (OR – Search within topic) and **Palliative Care** (OR – Search within topic) and **Maximum Conservative Management** (OR – Search within topic) and **Maximal Conservative Management** (OR – Search within topic) and **Conservative Management** (OR – Search within topic)

**And**

**renal replacement therapy** (Topic) and **Dialysis** (OR – Search within topic) and **Peritoneal Dialysis** (OR – Search within topic) and **Haemodialysis** (OR – Search within topic) and **Hemodialysis** (OR – Search within topic) and **Rrt** (OR – Search within topic) and **Krt** (OR – Search within topic) and **Kidney Replacement Therapy** (OR – Search within topic)

**Supplementary file 3: Adapted Newcastle-Ottawa Scale for cross-sectional analyses**

This scale has been adapted from the Newcastle-Ottawa Quality Assessment Scale for cohort studies to provide a quality assessment of cross-sectional studies. Allocate 1 point for every statement with an asterix.

**Selection: (Maximum 5 stars)**

1. Representativeness of the sample:

a. Truly representative of the average in the target population. * (all subjects or random sampling)

b. Somewhat representative of the average in the target group. * (e.g consecutive sampling)

c. Selected group of users/convenience sample.

d. No description of the sampling strategy.

2. Sample size:

a. Justified and satisfactory (> 100 participants). *

b. Not justified/ insufficient.

3. Non-respondents:

a. The response rate is satisfactory and there is limited missing data. *

b. The response rate is unsatisfactory and/or a large amount of data is missing.

c. The response rate is not reported.

4. Ascertainment of exposures:

a. Collected prospectively from participants, clinicians or the electronic health record. **

b. Collected retrospectively (e.g. from review of electronic health records), but methods clearly described and justified *

c. measurement methods not described

**Comparability: (Maximum 2 stars)**

1. Comparability of subjects in different outcome groups based on design or analysis.

a. Outcome groups selected from the same source population (e.g. new referrals to an advanced CKD clinic).**

b. Information was not provided or groups were not comparable.

**Outcome: (Maximum 3 stars)**

1. Assessment of the outcome (Choosing renal supportive care)

a. Reported by participant or clinician . **

b. Based on formal documentation in electronic health record. **

c. Status assumed based on absence of dialysis record. *

d. Information not provided.

2. Statistical test:

a. Statistical test used to analyse the data clearly described, appropriate, and measures of association presented including confidence intervals and probability level (P value). *

b. Statistical test not appropriate, not described, or incomplete.

| **Supplementary file 4: Table showing summary of modified Newcastle-Ottawa Scale score for risk of bias** | | | | |
| --- | --- | --- | --- | --- |
| **Study** | **Selection (total = 5)** | **Comparability (total = 2)** | **Outcome (total = 3)** | **Total (total = 10)** |
| **Australian Studies (n = 4)** | | | | |
| Brown et al | 4 | 2 | 2 | 8 |
| Chou et al | 4 | 2 | 2 | 8 |
| Morton et al | 5 | 2 | 3 | 10 |
| So et al | 3 | 0 | 2 | 5 |
| **United Kingdom Studies (n = 11)** | | | | |
| Carson etc a | 4 | 2 | 2 | 8 |
| Chandna et al | 4 | 2 | 2 | 8 |
| Chanouzas et al | 3 | 2 | 2 | 7 |
| Chess et al | 4 | 2 | 2 | 8 |
| Hussain et al | 4 | 2 | 2 | 8 |
| Pyart et al | 4 | 2 | 3 | 9 |
| Raman et al | 3 | 2 | 2 | 7 |
| Rosenberg et al | 3 | 2 | 3 | 8 |
| Murtagh et al | 4 | 2 | 2 | 8 |
| Shah et al | 4 | 0 | 2 | 6 |
| O’Keefe et al | 4 | 2 | 2 | 8 |
| **North American Studies (n = 3)** | | | | |
| Scherer et al | 4 | 2 | 3 | 9 |
| Wong et al | 3 | 2 | 3 | 8 |
| Tam-Tham et al | 4 | 2 | 1 | 7 |
| **European Studies (n = 13)** | | | | |
| Reindl-Schwaighofer et al | 4 | 0 | 2 | 6 |
| Moranne et al | 4 | 2 | 2 | 8 |
| Joly et al | 4 | 2 | 2 | 8 |
| Martino et al | 2 | 2 | 2 | 6 |
| van Loon et al | 5 | 2 | 2 | 9 |
| Verberne et al | 4 | 2 | 2 | 8 |
| Arenas et al | 3 | 2 | 2 | 7 |
| Garcia et al | 3 | 2 | 2 | 7 |
| Guerrero Riscos et al | 5 | 2 | 2 | 9 |
| Villareal et al | 2 | 2 | 3 | 7 |
| Teruel et al | 4 | 2 | 3 | 9 |
| Guitierrez Sanchez et al | 3 | 0 | 2 | 5 |
| Garcia Garcia et al | 3 | 0 | 2 | 5 |
| **Hong Kong Studies (n = 5)** | | | | |
| Chan et al | 4 | 2 | 2 | 8 |
| Kwok et al | 4 | 2 | 2 | 8 |
| Shum et al | 4 | 2 | 2 | 8 |
| Yong et al | 5 | 0 | 2 | 7 |
| Yuen et al^42^ | 4 | 2 | 2 | 8 |
| **South East Asian Studies (n = 5)** | | | | |
| Wan et al | 3 | 2 | 2 | 7 |
| Seow et al | 3 | 2 | 2 | 7 |
| Noppakun et al | 4 | 2 | 2 | 8 |
| Teo et al | 4 | 0 | 2 | 6 |
| Ch’ng et al | 3 | 2 | 0 | 5 |
| **African Studies (n = 2)** | | | | |
| Mathew et al^46^ | 5 | 0 | 1 | 6 |
| Okyere et al^47^ | 4 | 0 | 3 | 7 |

**
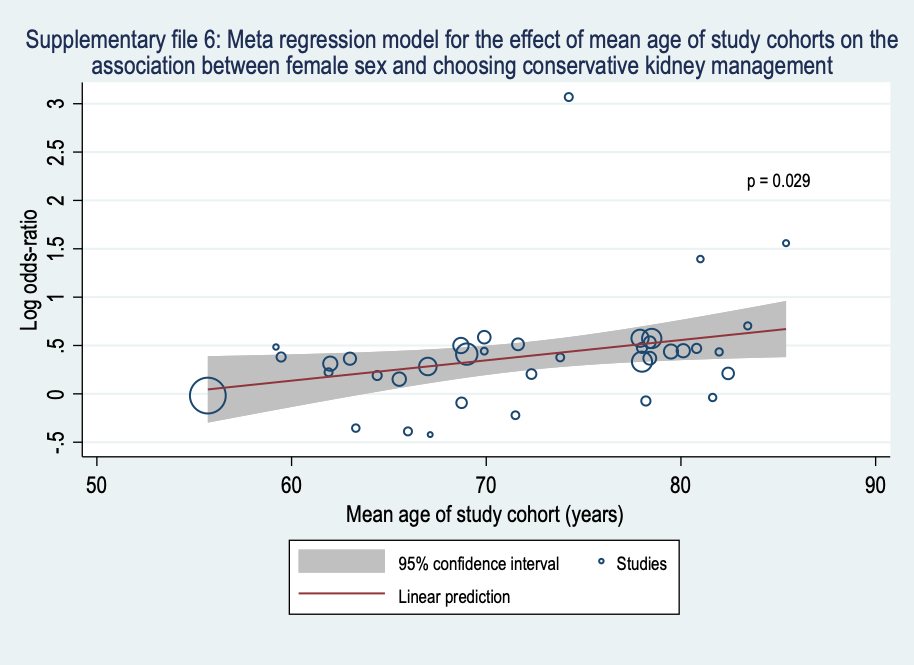
**

**
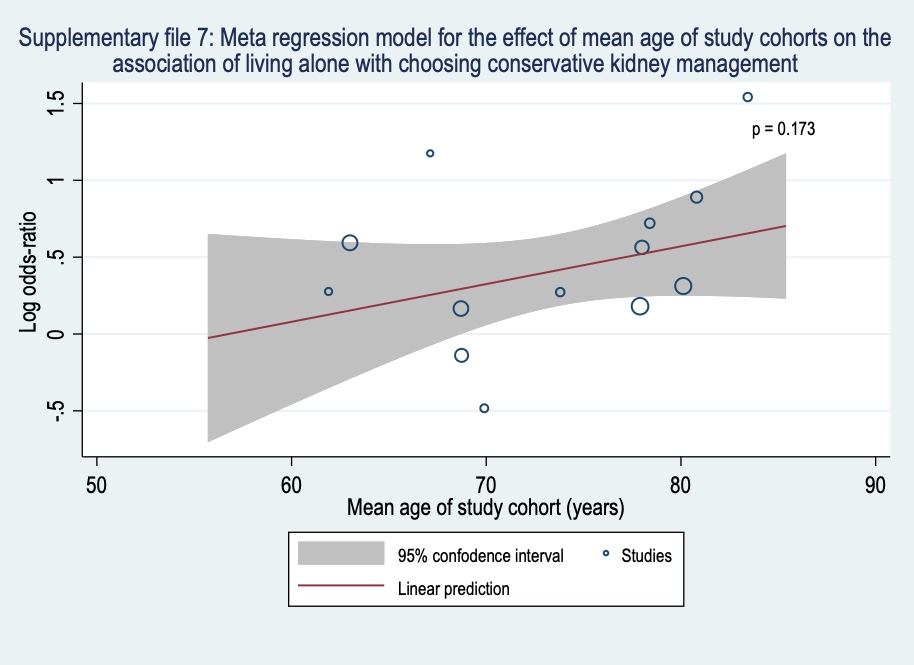
**
